# The Anti-Tuberculosis War and the Red Cross Christmas Stamp—An Appeal

**Published:** 1910-01

**Authors:** S. Adolphus Knopf

**Affiliations:** Professor of Phthisio-therapy at the New York Post Graduate Medical School and Hospital


### The Anti-tuberculosis War and The Red Cross Christmas Stamp—An Appeal.

By S. ADOLPHUS KNOPF, M. D.,
Professor of Phthisio-therapy at the New York Post Graduate Medical School and Hospital.

LAST fall it was my privilege to address the two Red Cross branches,—one in Brooklyn and one in New York,—pleading with them to help in the anti-tuberculosis war through the aid of a Red Cross Christmas stamp. I published the two addresses in the form of an article in the *New York Medical Journal* of November 28, 1908. I know that hundreds of others, nay even thousands, have also pleaded, and perhaps more eloquently and more successfully than I; but this shall not prevent me from pleading again for this holy cause, particularly since I have been honored by the officers of the American National Red Cross with an invitation to do so.

The history of the Red Cross is known to most people. It owes its origin to the feeling of sympathy awakened throughout Europe by the sufferings occasioned by the Crimean war. The object of the Red Cross Society is in the main to mitigate the evils inseparable from war. All of the civilised nations of the world have branches of this truly international association. Founded in Geneva in 1863, it is now not quite fifty years old, but what a glorious work it has done! Throughout the many bloody wars of the last half century the Red Cross servants were truly the administering angels who lessened suffering and saved countless lives. And not only in wars, but also in other disasters such as floods, earthquakes, mining and railroad accidents, fires and pestilences, a great army of Red Cross soldiers are always present to ameliorate conditions, dress the wounded, nurse the sick, feed the hungry and improve sanitation so as to limit the fatalities as much as may be possible. The heroism of the Red Cross workers, both men and women, has never been surpassed by the gallantry of the bravest soldiers.

Now, this great association has undertaken to fight the most formidable enemy of mankind; one which unfortunately can not be met openly in battle; one which, by its insidiousness and because it is unseen and unrecognised by the naked eye, is all the more dangerous and difficult to combat. There are probably at this moment 500,000 people in the United States suffering from tuberculosis in one form or another, and 1,000,000 school children who are probably destined to die of tuberculosis before they reach the age of eighteen, and yet modern medical science has demonstrated beyond a shadow of a doubt that tuberculosis is a preventable and curable disease.

Its prevention depends upon bettering the hygiene of the masses and improving their living conditions, on the early recognition of the disease, and on the suppression of all centers of infection arising from advanced cases. This is to be accomplished not with cruel isolation or treating the unfortunate consumptive as an outcast, but by removing the consumptive poor to special hospitals where they will be kindly treated and the utmost care exercised to improve their condition and at the same time minimise the danger of infecting others. The home of the conscientious well-to-do consumptive, in the advanced stages, can be arranged so that there is really no danger of contagion.

The cure of the tuberculous depends upon the early recognition of the disease and the timely treatment in well arranged sanitary homes or in special institutions, sanatoria, hospitals, or camps, and there is urgent need for such institutions in nearly every state of the union. Of course, for the tuberculous children we must have many open-air schools and children's sanatoria; and for the tuberculous adult, cured or sufficiently improved to do some work, we must have agricultural or horticultural colonies or other means to give him outdoor occupation.

Unfortunately, tuberculosis is a disease which is most prevalent among the poor, and after what has been said I need not explain any further that in order to prevent and cure tuberculosis in our own beloved country, we need a great deal of money. All the skill of the physician and the devotion of the nurse is of no avail when the tuberculous patient lacks the means to buy good food, cannot afford to live in a sanitary home, have proper clothing, or rest when rest is his only salvation. The patient's anxiety for those depending upon him must also be removed. The wife or children, the aged father or mother deprived of their supporter must be cared for. Tranquility of mind is as essential to the cure of tuberculosis as all other factors. To do all this, I say again we need money, much money.

Fortunately, this country is rich and it does not lack in philanthropy and brotherly love, and I know that this appeal which is now going out from the Red Cross will not be in vain. It will give opportunity to the humblest of the humble, to the richest among the rich, to help in this great, good and holy cause of saving lives, making tuberculous children into strong and healthy citizens, the curable consumptives into breadwinners for their families, and rendering the hopelessly ill consumptive comfortable and happy as far as it is in human power to do.

The whole nation will reap the benefit of a successful war against tuberculosis and this benefit will not only be sanitary and moral but even financial, for every restored breadwinner and healthy citizen is an addition to the wealth of the nation.

But let us put aside for a moment the financial aspect. Christmas-tide is not a season when we calculate on returns for what we give. We find pleasure and delight in giving, in making others happy, and surely here is a splendid opportunity to do this. Let each one buy as many stamps as he can; tell the little children that every penny they can spare for stamps will help to save a little child's life, and although they may not see the little sufferer and receive direct thanks, they as well as the adults can rest assured that their gifts will be appreciated and the unknown donor remembered in the grateful prayers of some tuberculous invalid.

The 1909 Red Cross Christmas stamp is not good for postage. It will not carry any kind of mail but any kind of mail will carry it. The use of the beautiful Red Cross stamp carrying Christmas and New Year's greetings, gives an excellent opportunity to everyone to help the anti-tuberculosis cause according to his means. The layman will thus be the co-worker of the physician, a true brother and helper. He who makes his Christmas offering by the purchase of as many of these stamps as he can afford to buy will surely feel the season's joy all the more, knowing that through his participation in this work somewhere some consumptive sufferer has been helped, some dark home made brighter, some little child saved.

